# Missed opportunities for vaccination among children aged 0–23 months visiting health facilities in a southwest State of Nigeria, December 2019

**DOI:** 10.1371/journal.pone.0252798

**Published:** 2021-08-27

**Authors:** Akinola Ayoola Fatiregun, Laura Nic Lochlainn, Lassané Kaboré, Modupeola Dosumu, Elvis Isere, Itse Olaoye, Francis Adegoke Akanbiemu, Yetunde Olagbuji, Rosemary Onyibe, Kofi Boateng, Richard Banda, Fiona Braka

**Affiliations:** 1 World Health Organization Ondo State Office, Akure, Nigeria; 2 World Health Organization Headquarter, Geneva, Switzerland; 3 Faculty of Medicine, Institute of Global Health, University of Geneva, Geneva, Switzerland; 4 Ondo State Primary Health Care Development Agency, Ministry of Health, Akure, Ondo State, Nigeria; 5 World Health Organization Southwest Zonal Office Ibadan, Ibadan, Nigeria; 6 World Health Organization Country Office, Abuja, Nigeria; All India Institute of Medical Sciences Bhopal, INDIA

## Abstract

**Background:**

Despite efforts to improve childhood immunization coverage in Nigeria, coverage has remained below the national acceptable level. In December 2019, we conducted an assessment of Missed Opportunities for Vaccination (MOV) in Ondo State, in Southwest Nigeria. The objectives were to determine the magnitude of, explore the reasons for, as well as possible solutions for reducing MOV in the State.

**Methods:**

This was a cross-sectional study using a mixed-methods approach. We purposively selected 66 health facilities in three local government authorities, with a non-probabilistic sampling of caregivers of children 0–23 months for exit interviews, and health workers for knowledge, attitudes, and practices (KAP) surveys. Data collection was complemented with focus group discussions and in-depth interviews with caregivers and health workers. The proportion of MOV among children with documented vaccination histories were determined and thematic analysis of the qualitative data was carried out.

**Results:**

984 caregivers with children aged 0–23 months were interviewed, of which 869 were eligible for inclusion in our analysis. The prevalence of MOV was 32.8%. MOV occurred among 90.8% of children during non-vaccination visits, and 7.3% during vaccination visits. Vaccine doses recommended later in the immunization series were given in a less timely manner. Among 41.0% of health workers, they reported their vaccination knowledge was insufficient. Additionally, 57.5% were concerned about and feared adverse events following immunization. Caregivers were found to have a low awareness about vaccination, and issues related to the organization of the health system were found to contribute towards MOV.

**Conclusions:**

One in three children experienced a MOV during a health service encounter. Potential interventions to reduce MOV include training of health workers about immunization policies and practices, improving caregivers’ engagement and screening of vaccination documentation by health workers during every health service encounter.

## Introduction

Improving the coverage and equitable access to immunization as an entry point to primary health care (PHC) in Nigeria is a major objective of the National Primary Health Care Development Agency (NPHCDA). One of the strategic objectives of the Nigeria comprehensive Multiyear Plan (cMYP) 2016–2020 was to ensure that by 2020, 95% of infants, in at least 90% of districts were fully immunized against vaccine-preventable diseases before reaching 12 months of age [1]. Since 2013, there has been considerable progress in routine immunization performance in the country. However, findings from the 2016/17 National Immunization Coverage Survey (NICS) indicated that the strategic objectives on coverage and equity were far from being achieved [2].

In Ondo State, one of the six states in Southwest Nigeria, routine immunization coverage over the past two years has stagnated. Based on results of trends from the Districts Health Information System (DHIS-2) platform, NICS, Multiple Indicator Coverage Survey (MICS) and Lots Quality Assurance Sampling Survey (LQAS) immunization coverage is below the national and global acceptable level of ≥ 90% [2]. Furthermore, differences in immunization coverage from both administrative and survey data have been found for vaccines that should be administered during the same visit according to the national immunization schedule, suggesting some opportunities to deliver all due vaccines have been missed. Research aimed at evaluating the determinants of uptake for completing the Pentavalent and Oral Polio Vaccine (OPV) series found a 20% difference in the completion rate, indicating MOV had occurred since both vaccines are administered at the same time [3] Nationally, a 2.3% difference in coverage has also been found between yellow fever and measles while in Ondo State, it is 3.7% [2].

Among the reasons given by caregivers for their children not being fully immunized, 25% are linked to inequity in service delivery distribution points with, 12% reporting that immunization sites are too far [2,4]. Although missed opportunities for vaccination (MOV) have been described as one of the obstacles to raising immunization coverage among children [5,6], the magnitude and root causes of MOV as a contributing factor to the stagnated coverage have not been adequately evaluated in Ondo State.

A MOV includes any contact with health services by a child (or adult) who is eligible for vaccination (unvaccinated, partially vaccinated or not up-to-date, and free of contraindications to vaccination), but which does not result in the individual receiving all the vaccine doses for which he or she is eligible [5–8]. Based on previous MOV assessments, MOV can occur due to health system, health worker or caregiver related issues [5–18]. As part of the World Health Organization (WHO) Scholar course “Reducing inequities and improving coverage”, a successful proposal was developed in May 2019 for the assessment of MOV in Ondo State. In December 2019, the Ministry of Health and immunization partners, participated in the MOV assessment led by WHO. The objectives of the MOV assessment were to determine the magnitude of MOV among children 0–23 months visiting health facilities, identify the underlying causes of MOV and explore what can be adjusted or done differently to reduce missed opportunities and improve vaccination coverage and equity.

## Methods

### Study design and setting

This was a cross-sectional study that employed a mixed-methods approach, incorporating both qualitative and quantitative tools for data collection in line with the WHO MOV methodology (Table 1) [5]. The quantitative component of the assessment included exit interviews with caregivers of children <24 months and anonymous self-administered health worker knowledge, attitude and practices (KAP) surveys. The qualitative component included focus group discussions (FGDs) with caregivers, health workers and in-depth interviews (IDIs) with senior health facility staff or health administrators. The WHO MOV guides and past MOV assessments provide more detailed information on the assessment process and expected outcomes [5–18].

**Table 1:** Matrix for implementing the Missed Opportunities for Vaccination (MOV) Assessment, Ondo State, Nigeria, 2019.

MOV objectives	Assessment tool components	Types of data collected
Identify the magnitude and causes of missed opportunities for vaccination (MOV)	Health facility exit interviews for caregivers (interviewer-administered)	• socio-demographic information; • vaccination history (routine immunization)* • awareness of opportunities for routine immunizations; • vaccination card availability and retention; • reasons for non-vaccination; and • quality of the vaccination service received.
Understand the underlying causes of MOV and explore potential solutions	Health worker knowledge, attitudes, and practices survey KAP (interviewer-administered)	• Knowledge about vaccination, including antigens, immunization schedules, and contraindications to vaccination.
	Focus group discussions (with mothers/caregivers and health workers)	• Exploring causes of MOV, and potential solutions and barriers to implementation of proposed interventions.
	In-depth interviews (with senior staff and health administrators)	• Interviews conducted with individuals who are insightful or influential about the health facility or the community, such as the health facility heads or in-charges, directors, matrons, and administrators. This facilitates triangulation with other data elements and provides information regarding the informants’ perception of the community and health facility dynamics affecting immunization services, informant support or opposition to immunization. • Key informants were asked to explain health worker and caregiver behaviors/responses and suggest or validate/refute previously proposed interventions to reduce missed opportunities.
Identify potential interventions to reduce MOV	Workgroup brainstorming sessions	• Present preliminary assessment findings to immunization stakeholders and formulate an action plan with proposed interventions.

* Vaccination status was determined from vaccination documentation (vaccination cards or health facility registers), where possible. The assessment did not rely on the caregivers’ recall if the required vaccination card was lost or otherwise unavailable. This is particularly important because the date of vaccination is critical in assessing MOV [4,5].

**Fig 1:**
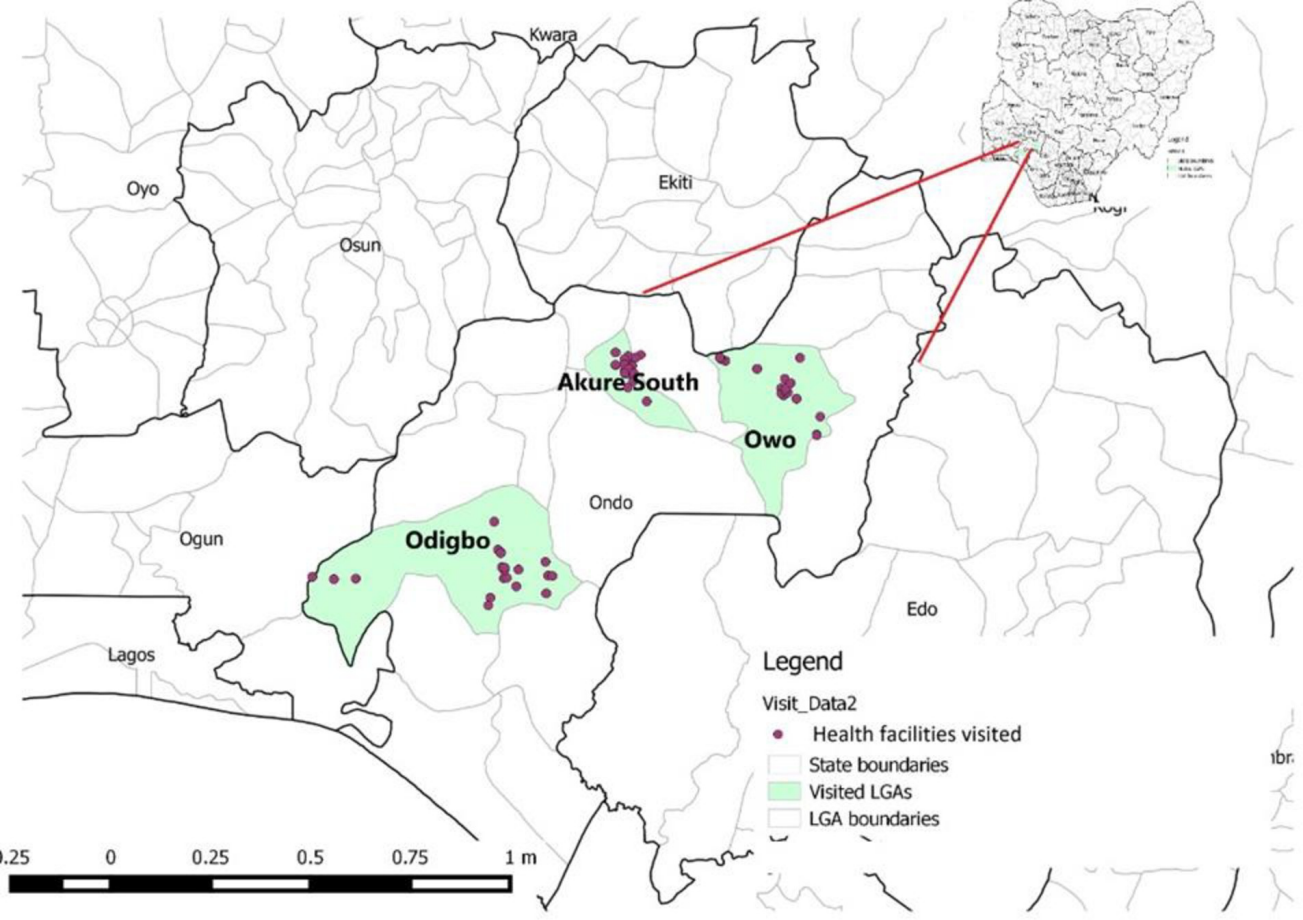
Map of Ondo State, Nigeria showing the selected local government areas (LGAs) and health facility locations for the 2019 Missed Opportunities for Vaccination (MOV) assessment.

The MOV assessment was carried out in three local government areas (LGAs) representing the three political senatorial districts in Ondo State (Fig 1) [19,20]. With close to 1000 public and private health facilities in Ondo State, an estimated 62% (618) are providing routine immunization services.

### Sampling criteria

We purposively selected one LGA from each of the three senatorial districts in Ondo State, Akure South from the central senatorial district, Owo from the north, and Odigbo from the southern district. The selected LGAs are the largest within each senatorial district, with 50–60% of the political wards having rural settlements, and representing the different ethnic subgroups found within the districts. The 66 health facilities (public (53) and private (13)), that were listed in the DHIS-2 database as conducting routine immunization services in the selected LGAs were visited for the caregivers’ exit interviews, health workers KAP survey, as well as IDIs and FGD sessions. Fifty-one health facilities operate at the primary healthcare level, 13 at secondary healthcare level and two at tertiary healthcare level.

### Study procedures

Preparation for fieldwork and pilot testing of the data collection tools commenced in November 2019. Facilitators (WHO Ondo State office personnel) finalized the logistical details for the assessment, which included compiling the list of sampled facilities, transport arrangements, training, as well as data collection tools.

The WHO MOV training materials were adapted and used [16] to train the field teams which comprised of 50 (33 females and 17 males) data collectors and 12 (7 females and 5 males) supervisors. The data collectors were Master’s of Public Health (MPH) students and graduates with previous experience in mixed methods (qualitative and quantitative) approaches to data collection, analysis and interpretation. The teams were centrally trained for two days on the MOV methodology and strategy in order to prepare them for four days of data collection using the electronic data platform OpenDataKit (ODK).

Following the field work, a one-day brainstorming session was held with the field teams, representatives from the Ministry of Health, and immunization partners. A work plan was developed with a chronogram of activities, including a supervision evaluation and monitoring plan, budget, and budget sources, as well as responsible persons or organization. A briefing and debriefing was then held with Ondo State Primary Health Care Board (OSPHCB) whereby justification, objectives, methodology, and plan for implementation of the work plan were presented and thoroughly discussed.

### Data collection tools

Data collection tools were adapted using the MOV standardized quantitative and qualitative tools (Table 1) pretested and deployed using the ODK application. For the quantitative tools, the caregiver health facility exit interview questions included: socio-demographic information; child’s vaccination history (received through routine immunization only); awareness of opportunities for routine immunizations; vaccination documentation availability and retention; reasons for non-vaccination; and feedback on the quality of the vaccination services received.

Health worker KAP surveys included socio-demographic information; knowledge and attitudes toward vaccination; additional questions on vaccination practices and decision-making for health workers whose regular duties included administering vaccines. Both questionnaires included core questions, as well as additional questions, and single and multi-select responses. All questionnaires were written in English.

The qualitative data collection tools included semi-structured FGD guides for caregivers and health workers and IDI guides for key informant interviews. The guides included opening questions, followed by key questions which focussed on the health and vaccination services in the community, attitudes toward vaccination and vaccination compliance, reasons for MOV and suggestions to reduce MOV. The guides ended with closing questions to summarize the discussion or interview.

### Data collection

Data were collected using the ODK application. The forms were accessible with a specified URL, username and password. The field teams spent three days collecting health facility caregiver exit interviews, health workers KAP and IDIs, and one day conducting caregiver and health worker FGDs.

To determine vaccination history, photos of child vaccination documentation (home-based records or health facility registers (when vaccination cards were not found)) were taken and uploaded to the ODK application. These photos were later reviewed for validation purposes. If the vaccination documentation was unavailable, the field teams did not rely on caregiver recall about vaccination history.

The field teams conducted exit interviews as caregivers were leaving the health facility after receiving health services. A target sample size of 600 caregiver exit interviews and 300 health worker questionnaires was determined, as recommend by WHO MOV methodology [5,6]. Therefore, at each health facility, the field teams aimed to conduct 20 sequential exit interviews with consenting caregivers of children 0–23 months old in the morning and 10 health workers KAP surveys in the afternoon, once services were completed. The IDIs were conducted on purposive samples of one senior staff or health administrators per health facility.

Field teams included caregivers of children attending a health facility that offers routine immunization services in one of the selected districts on an assessment day. Regardless of the reason for visiting the health facility, their place of residence or relationship to the child, all caregivers were eligible to be interviewed if they were aged at least 18 years. If a caregiver was accompanied by more than one child, the interview was focussed on the youngest child. For health workers KAP surveys, we included staff from both preventive and curative departments who were at the selected health facilities.

Caregiver and health worker FGD sessions were held separately, with 6–10 participants per FGD. These were conducted at six health facilities that were not selected for caregivers and health workers interviews. The ODK platform was used to record and upload voice recordings of IDI and FGD responses. The FGD participants were purposively selected and they did not participate in the quantitative data collection. The FGD sessions were conducted in both English and Yoruba, the local language. The transcription, translation and back translation of the local language was done by the facilitators and the FGD data collectors to ensure standardization. Each FGD and IDI session spanned an average of 65mins and 45mins respectively. Themes were explored until additional discussions did not lead to any new or emerging themes. Transcripts were not returned to participants, and no repeat interviews were conducted. The ODK application automatically recorded the time, duration of the interviews, as well as the location where they were completed. Supervisors reviewed data collection forms for completeness and accuracy before submission to the server. Interviewers uploaded data every evening, and further data quality checks were conducted. To enable quality of data entry, the data entry forms were designed with value constraints, to the extent possible.

### Data management and analysis

The caregiver exit interviews and health worker KAP data were downloaded from the ODK server and exported to Microsoft Excel, for data cleaning, data management, and analysis.

Frequency distributions of socio-demographic and other relevant variables from the caregiver exit interviews and the health workers’ KAP were analysed. Following the standard methodology used in the analysis of previous MOV assessments [5–18], we created a flow chart to identify children with MOV (Fig 1). We created frequency distributions for children with documented vaccination history, and those eligible for one or more vaccine doses during their health facility visit. We calculated MOV based on the child’s date of birth and interview date, the national immunization schedule (Table 2), and if they had contraindications to vaccination on the assessment day (as reported by the caregiver). Only children who were eligible for one or more vaccine dose on the assessment day and with documented vaccination history were included in the calculation of MOV. We distinguished between children who received all eligible doses, some, but not all the doses, and no doses during the health facility visit. We estimated the proportion of dose-based MOV, which we defined as the number of missed doses as a proportion of the total number of eligible doses that would have been given if there had been no MOV. We also examined the correlation between MOV and other key variables.

**Table 2:** Classification of timeliness of vaccinations received by children aged 0–23 months surveyed during a Missed Opportunities for Vaccination (MOV) assessment by time intervals, using the national schedule on immunization, Ondo State, Nigeria, 2019.

Vaccine	Schedule age of vaccination	Too early	Timely	Delayed
**Birth dose**				
BCG^1^	Birth	-	0–30 days	30–365 days
OPV^2^			0–14 days	14–365 days
Hepatitis B vaccine			0–14 days	14–365 days
**First dose**	6 weeks (42 days)	<42 days	42–56 days	≥ 57 days
OPV				
Pentavalent vaccine^3^				
PCV				
**Second dose**	10 weeks (70 days)	<70 days	70–84 days	≥84 days
OPV				
Pentavalent vaccine				
PCV				
**Third dose**	14 weeks (98 days)	<98 days	98–112 days	≥ 113 days
OPV				
Pentavalent vaccine				
PCV				
IPV				
**Measles-1**	9 months (270 days)	<270 days	270–365 days	≥ 365 days
**Yellow Fever**	9 months (270 days)	<270 days	270–365 days	≥ 365 days
**Meningitis A**	9 months (270 days)	<270 days	270–365 days	≥ 365 days
**Measles-2**	15 months (450 days)	< 450 days	450–690 days	>690 days*

^1^ Bacille Calmette-Guerin (BCG) vaccine.

^2^ Oral Polio Vaccine (OPV).

^3^ Diphtheria-tetanus-pertussis-hepatitis B-Haemophilus influenza type b (pentavalent) vaccine.

To determine the timeliness of vaccination for each antigen, we calculated the interval between the date of birth and date of vaccination in days and used the national immunization schedule for the expected time intervals between each antigen (Table 2). We defined a vaccination as early if it was given before the expected interval, timely when the calculated interval fell within the expected interval in the schedule; and delayed if the calculated interval was after the expected interval.

The IDI and FGD responses were downloaded as media files and the compilation of notes taken were transcribed for analysis. Thematic analyses of the qualitative data were conducted using Atlas TI software. These are presented as observations and representative verbatim quotes. Four data coders coded, categorised and analysed the data. Seven major themes from the IDI and FGD guides were; community perception about vaccination, compliance with vaccination, satisfaction with vaccination, reasons for incomplete vaccination, non-compliance and missed opportunities, challenges to vaccination and strategies for improvement of vaccination were generated from transcribed interviews. The major themes had 24 sub-themes and 60 codes. Minor themes were identified and described. The qualitative and quantitative data were triangulated at each stage of data collection, analysis and interpretation, as outlined in the MOV methodology [5,6].

### Ethical considerations

Ethical approval was obtained from the Ondo State Health Research Ethics Committee (Protocol Number: OSHREC/13/12/19/243). Also, verbal informed consent was obtained from the respondents by the interviewers following the provision of information about the assessment and its objectives. Respondents were also informed that participation was voluntary and there was no consequence for non-participation. There was minimal collection of personally identifiable information from caregivers and health workers, and all information obtained were kept confidential on a password protected server.

## Results

The MOV assessment was completed within 10 days, including training of field teams, data collection, preliminary data analysis, and a brainstorming session. A total of 984 caregiver exit interviews, 332 health worker KAPs were collected from 42 health facilities across the three LGAs. Of the 984 caregiver exit interviews, 115 were excluded from the MOV analysis as they were missing the date of birth or vaccination documentation. For the qualitative data, six caregiver and six health worker FGD sessions were performed and 15 IDIs with health administrators.

### Caregiver and children demographics

Almost all caregivers were mothers (98.6%; 857/869) who had some education (94.1%). The child sex ratio was 1:1 and most children (79.1%; 687/869) were less than 12 months (Table 3). Less than half of the caregiver’s visits (44.8%; 389/869) were for vaccination. Vaccination status was validated by examining photos of vaccination cards (89.5%; 778/869) and health facility based registers (10.5%; 91/869). There were no statistically significant differences in the demographic characteristics of children or caregivers of those with validated documents used in the estimation of MOV and those who were excluded.

**Table 3:** Characteristics of caregivers of children 0–23 months surveyed during a Missed Opportunities for Vaccination (MOV) assessment with valid documented dates of birth and vaccination (n = 869), and those without (n = 115), Ondo State, Nigeria, 2019.

	Caregivers with documented evidence of vaccination n(%)	Caregivers without documented evidence of vaccination or date or birth n(%)	Total N(%)	P-value
	**869**	**115**	**984**	
***Child demographics***				
**Sex**	**869**	**115**	**984**	
Male	438(50.4)	62(53.9)	500(50.8)	
Female	431(49.6)	53(46.1)	484(49.2)	0.4792
**Age**	**869**	**115**	**984**	
0–11 months	687(79.1)	97(84.3)	784(79.7)	
12–23 months	182(20.9)	18(15.7)	200(20.3)	0.1832
***Caregivers demographics***				
**Caregivers relationship with the child**	**869**	**115**	**984**	
Mother	857(98.6)	110(96.5)	967(98.3)	
Father	2(0.2)	2(1.8)	4(0.4)	
Others (grandparent, aunt/uncle, brother etc.)	10(1.2)	2(1.8)	12(1.2)	CCNM^3^
**Educational Level**	**869**	**115**	**984**	
Completed primary	172(19.8)	26(22.6)	198(20.1)	
Completed secondary	422(48.6)	57(49.6)	479(48.7)	
Did not complete primary (less than 6 years)	28(3.2)	3(2.6)	31(3.2)	
More than secondary	184(21.2)	21(18.3)	205(20.8)	
No formal education	51(5.9)	5(4.3)	56(5.7)	
Others	12(1.4)	3(2.6)	15(1.5)	0.7954
***Health facility visit***				
**Type of health facility**	**869**	**115**	**984**	
Private for-profit	32(3.7)	13(11.3)	45(4.6)	
Private not for profit	1(0.1)	1(0.9)	2(0.2)	
Public/Government service	836(96.2)	101(87.8)	937(95.2)	CCNM^3^
**Vaccination card availability (on day of visit)**	**869**	**115**	**984**	
Yes, and I have it with me	778(89.5)	87(75.7)	865(87.9)	
Yes, but I do not have it with me	91(10.5)	27(23.5)	118(12.0)	
No	0(0)	1(0.9)	1(0.1)	CCNM^3^
**Health worker asked for child vaccination card**	**869**	**115**	**984**	
Yes	621(71.5)	91(79.1)	712(72.4)	
No	248(28.5)	24(20.9)	272(27.6)	0.0808
**Reason for visit**	**869**	**115**	**984**	
Vaccination visit	389(44.8)	47(40.9)	436(44.3)	
Medical consultation	93(10.7)	5(4.3)	98(10.0)	
Healthy child visit or check-up	228(26.2)	36(31.3)	264(26.8)	
Child was accompanying adult	147(16.9)	21(18.3)	168(17.1)	
Hospitalization	4(0.5)	3(2.6)	7(0.7)	
Other	8(0.9)	3(2.6)	11(1.1)	CCNM^3^
***Perception of immunization***				
**How would you assess your level of knowledge about vaccines/vaccination?**	**869**	**115**	**984**	
Vaccines prevent diseases so children will grow up healthy	841(96.8)	113(98.3)	954(97.0)	
Not sure what vaccines are for	27(3.1)	2(1.7)	29(2.9)	
Others	1(0.1)	0(0)	1(0.1)	0.6703
**Ever requested but refused vaccination services?**	**869**	**115**	**984**	
Yes	35(4.0)	1(1.8)	36(3.7)	
No	834(96.0)	114(98.2)	948(96.3)	0.0728
**Ever asked to pay for vaccines?**	**869**	**115**	**984**	
Yes	7(0.8)	0(0)	7(0.7)	
No	862(99.2)	115(100)	977(99.3)	0.4178
**Ever asked to pay for a child vaccination card?**	**869**	**115**	**984**	
Yes	18(2.1)	0(0)	18(1.8)	
**No**	851(97.9)	115(100)	966(98.2)	0.1046
**Informed about vaccination reactions?** ^**1**^	**401**	**45**	**446**	
Yes	329(82.0)	42(93.3)	371(83.2)	
No	72(18)	3(6.7)	75(16.8)	0.0549
**Informed about the next vaccination?** ^**1**^	**401**	**45**	**446**	
Yes	364(90.8)	44(97.8)	408(91.5)	
No	37(9.2)	1(2.2)	38(8.5)	0.1105
**Satisfied with today’s vaccination service?** ^**1**^	**401**	**45**	**446**	
Yes	396(98.8)	44(97.8)	440(98.7)	
No	5(1.2)	1(2.2)	6(1.3)	0.5902
**Suggestions for improving health facility services** ^**2**^	**291**	**32**	**323**	
Less of wait time	101(34.7)	3(9.4)	104(32.2)	0.0021
More personnel should be available	113(38.8)	11(34.4)	124(38.4)	0.6358
Hours and days for vaccination should not be limited	35(12.0)	1(3.1)	36(11.2)	0.1204
Vaccines should always be in stock	60(20.6)	8(25.0)	68(21.1)	0.5589
Vaccination should remain free	93(32.0)	16(50)	109(33.8)	0.0482
Information should be provided on vaccines given, diseases they prevent and reactions vaccines can produce	32(11.0)	4(12.5)	36(11.2)	0.7661
More outreach sessions	60(20.6)	3(9.4)	63(19.5)	0.1229
More friendly treatment of caregivers	14(4.8)	2(6.3)	16(5.0)	0.6877^4^

^1^ Among those who reported having been vaccinated on the day of health facility visit.

^2^ Multiple responses allowed.

^3^ One or both of the Cochran criteria for accepting chi-square values: (1) No more than 20% of cells have expected < 5. (2) No cell has an expected value < 1, not met. Cochran Criteria Not Met (CCNM).

^4^ Fisher exact test.

### Prevalence of MOV among children aged 0–23 months in Ondo State

**Fig 2:**
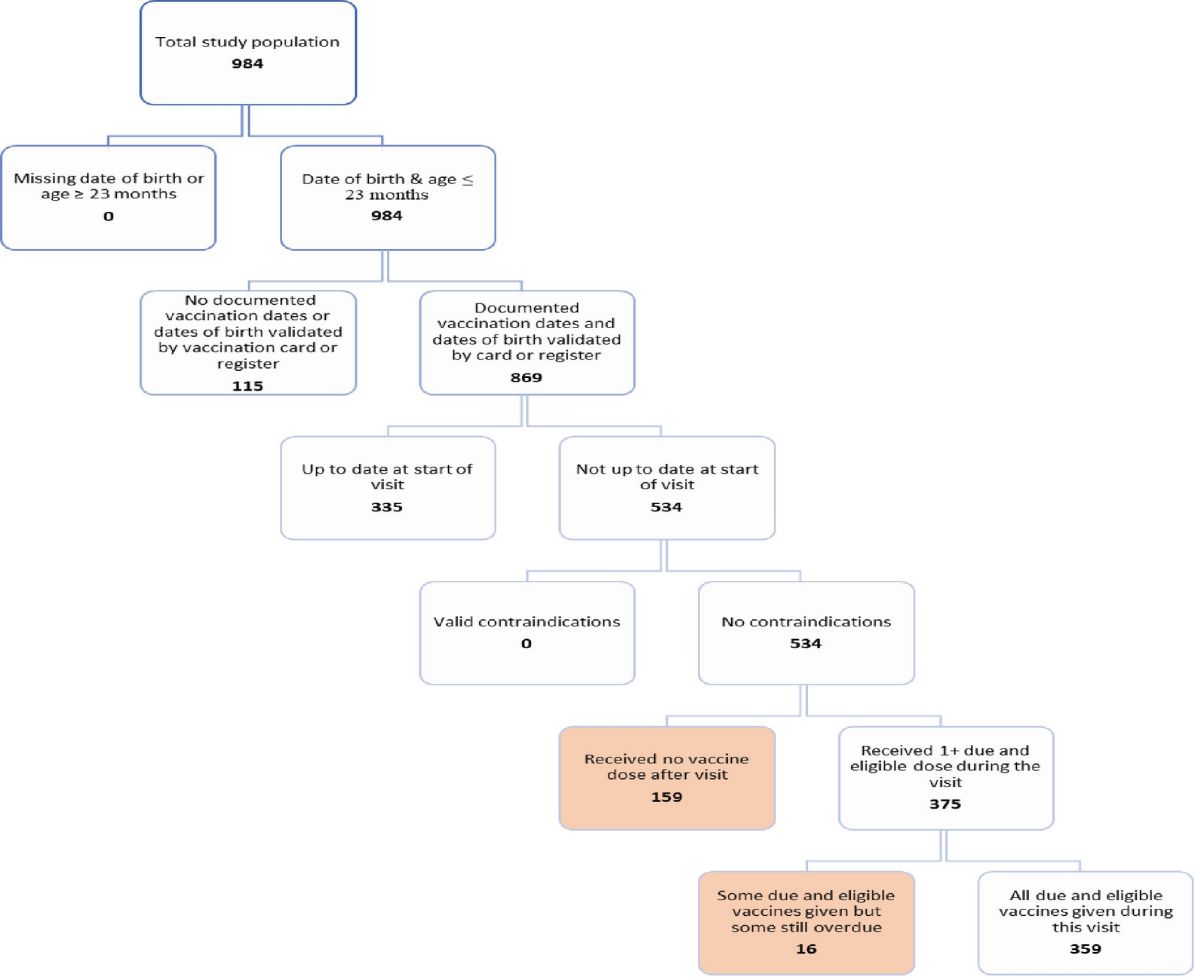
Health facility–based flow-chart for determining missed opportunities for vaccination (MOV)^1^, Ondo State, Nigeria, 2019. ^1^ Missed opportunity for vaccination (MOV): contact with health services by a child (or adult) who is eligible for vaccination (unvaccinated, partially vaccinated/not up-to-date, and free of contraindications to vaccination), which does not result in the individual receiving all the vaccine doses for which he or she is eligible.

Of the 869 children aged 0–23 months with valid vaccination documentation, 61.5% (534/869) were not up to date at the start of the visit, and had no valid contraindications to vaccination. However, only 70.2% (375/534) received at least one eligible dose they were due, while the remaining 29.8% (159/534) did not receive any due eligible dose(s). Among the 375 children who received at least one dose, 4.3% (16) did not receive all due doses. The proportion of MOV was 32.8% (175/534) (Fig 2).

### MOV by reason for visit

Among children at the health facility for a vaccination visit, 7.3% (27/371) experienced a MOV, compared with 90.8% (148/163) MOV for those attending for a non-vaccination visit. Medical consultation visits had a MOV prevalence of 96.8% (30/31), 94.9% MOV for a child accompanying an adult (56/59) and 86.6% MOV for healthy child visit or check-up (58/67) (Table 4).

**Table 4:** Prevalence of missed opportunities for vaccination (MOV)^1^ among surveyed children 0–23 months, by reason for visit, Ondo State, Nigeria, 2019.

	On arrival for health facility visit			During health facility visit		After health facility visit		
	Children with documented vaccination dates	Children with 1+ eligible doses due		Children vaccinated with all eligible doses during visit^2^		Children with 1+ MOV^2^		
	N	n	%	n	%	n	%	
Vaccination visit	389	371		344	92.7	27	7.3	
Non-vaccination visit	480	163	34.0	15	9.2	148	90.8	
*Medical consultation*	93	31	33.3	1	3.2	30	96.8	
*Healthy child visit or check-up*	228	67	29.4	9	13.4	58	86.6	
*Child is accompanying adult*	147	59	40.1	3	5.1	56	94.9	
*Hospitalization*	4	4	100.0	2	50.0	2	50.0	
*Other*	8	2	25.0	0	0.0	2	100	
**Total**	**869**	**534**	**61.5**	**359**	**67.2**	**175**	**32.8**	

^1^ Missed opportunity for vaccination (MOV): contact with health services by a child (or adult) who is eligible for vaccination (unvaccinated, partially vaccinated/not up-to-date, and free of contraindications to vaccination), which does not result in the individual receiving all the vaccine doses for which he or she is eligible.

^2^ Among the subset of children with documented vaccination dates and eligible for one or more vaccine doses (n = 534).

### Dose-based MOV and timeliness of vaccination

A total of 1489 doses were due during the health facility visits, of which 27.9% (416/1489) were missed. The highest proportion of missed vaccine doses were for meningitis A (MenA), and second dose of measles, with 54.4% (87/160) and 69.2% (54/78) of doses missed, respectively (Table 5).

**Table 5:** Timeliness of vaccination administered and missed opportunities by dose among children aged 0–23 months surveyed during a Missed Opportunities for Vaccination (MOV) assessment with documented vaccination histories, Ondo State, Nigeria, 2019.

Vaccine dose	Timeliness^1^				MOV dose		
	Total number of children who received dose^2^	Too early	Timely	Delayed	Eligible doses due	Eligible doses missed at visit	
	n	n (%)	n (%)	n (%)	n	n	%
**Birth dose**							
BCG^3^	859		750(87.3)	109(12.7)	69	8	11.6
OPV^4^	754		681(90.3)	73((9.7)	52	0	0
Hepatitis B	751		683(90.9)	68(9.1)	52	1	1.9
**First dose**							
OPV^4^	765	52(6.8)	578(75.6)	135(17.7)	109	22	20.2
Pentavalent ^5^	765	52(6.8)	577(75.4)	136(17.8)	109	22	20.2
PCV^6^	765	52(6.8)	577(75.4)	136(17.8)	116	22	19.0
**Second dose**							
OPV^4^	609	38(6.2)	431(70.8)	140(23.0)	80	9	11.3
Pentavalent ^5^	608	38(6.2)	431(70.9)	139(22.9)	93	17	18.3
PCV^6^	609	38(6.2)	431(70.8)	140(23.0)	87	11	12.6
**Third dose**							
OPV^4^	487	28(5.8)	306(62.8)	153(31.4)	86	31	36.1
Pentavalent^5^	487	28(5.8)	304(62.4)	155(31.8)	86	31	36.1
PCV^6^	487	28(5.8)	305(62.6)	154(31.6)	86	31	36.1
IPV^7^	479	28(5.9)	300(62.6)	151(31.5)	92	34	37.0
**Measles-1**	277	13(4.7)	254(92.0)	10(3.6)	67	18	26.9
**Yellow fever**	277	13(4.7)	254(92.0)	10(3.6)	67	18	26.9
**Meningitis A**	206	5(2.4)	128(62.1)	73(35.4)	160	87	54.4
**Measles-2**	74	9(12.2)	61(82.4)	4(5.4)	78	54	69.2
**Total**					1489	416	27.9

^1^ Please see Table 2 for intervals and immunization schedule used for this analysis.

^2^ Children with a documented history of receiving a dose either on the day of the survey or previously.

^3^ Bacille Calmette-Guerin (BCG) vaccine.

^4^ Oral poliovirus vaccine (OPV).

^5^ Diphtheria-tetanus-pertussis-hepatitis B-Haemophilus influenza type b (pentavalent) vaccine.

^6^ Pneumococcus conjugate vaccine (PCV).

^7^ Inactivated polio vaccine (IPV).

Timeliness of vaccination varied by vaccine and dose, with timely administration of vaccines ranging from 62.1% for MenA, to 92.0% for yellow fever and first dose of measles vaccines. The vaccine doses recommended in a series were given in a less timely manner; with timeliness of pentavalent vaccine decreasing from 75.4% for the first dose to 62.4% for the third dose (Table 5).

### Vaccination and caregiver attitudes

Among those whose children were vaccinated during the visit, most caregivers (90.8%; 364/401) stated that they were informed of their child’s next vaccination appointment dates and (82.0%; 329/401) of caregivers were told about potential adverse events following immunization (AEFI) (Table 3). Almost all caregivers (98.8%; 396/401) expressed their satisfaction with the vaccination services their children received. During a FGD, one participant said: “*What I see there is that I am satisfied with the vaccination services…I also see that the health workers here are hospitable*, *they don’t shout at us*, *they relate with us very well*, *and even play with us as well” FGD2_CG_06*.

Among those who made suggestions for improving services, more than a third (38.8%; 113/291) wanted more health workers in the health facilities; wanted less waiting time (34.7%; 101/291) and 32% (93/291) appealed that vaccination should remain free (Table 3).

### Health worker interviews

Health workers participating in the KAP surveys included Community Health Extension Workers (CHEW), Community Health Officers (CHOs), Junior Community Health extension workers (JCHEW), laboratory technicians, health assistants, nurses, and doctors. The highest proportion of health workers surveyed were community officers (48.7%) comprising of CHOs 6.0% (20/332), CHEW 34.0% (113/332), and JCHEWS 8.7% (29/332), while nurses and doctors accounted for 15.1% (50/332) and 3.0% (10/332), respectively (Table 6). Among the participating health workers, the majority (212/332) 63.9% had nine years or fewer clinical experience. Most (80.1%; 266/332) had previously been trained in vaccination or vaccine-preventable diseases.

**Table 6:** Characteristics and knowledge, attitudes, and practices of health workers surveyed during a Missed Opportunities for Vaccination (MOV) assessment, Ondo State, Nigeria, 2019.

	n	%
	332	
**Sex**	**332**	
Female	296	89.2
Male	36	10.8
**Professional training**	**332**	
Community Health extension workers (CHEW)	113	34.0
Community Health Officers (CHO)	20	6.0
Doctor	10	3.0
Junior Community Health extension workers (JCHEW)	29	8.7
Nurse	50	15.1
Others	110	33.1
**Years of experience**	**332**	
0–9 years	212	63.9
10–18 years	69	20.8
19–27 years	37	11.1
28 or more years	14	4.2
**Classification of health facility**	**332**	
Private for-profit	29	8.3
Private non-profit	4	1.1
Public/Government service	299	90.6
**Ever trained in vaccination or vaccine-preventable diseases?**	**332**	
Yes	266	80.1
No	66	19.9
***Health worker knowledge*, *attitudes*, *practices***		
**Contraindications for any vaccine** ^**1**^	**332**	
Local reaction to the previous dose	45	13.6
Light fever	104	31.3
Seizures under medical treatment	66	19.9
Pneumonia and other serious diseases	107	32.2
None of the above	108	32.5
**I feel my knowledge of vaccination is insufficient or out of date**	**332**	
Agree	136	41.0
Disagree	196	59.0
**I am concerned and fear Adverse Events Following Immunization (AEFI)?**	**332**	
Agree	191	57.5
Disagree	141	42.5
**When should vaccination status be assessed?** ^**1**^	**332**	
Child’s wellness/routine visit	141	42.5
Consultation for any illness	148	44.6
When a child is accompanying caregiver during a prenatal checkup	78	23.5
When a child is accompanying an adult for any reason	88	26.5
All of the above	134	40.4
**Why is vaccination status incomplete for some children?** ^**1**^	**332**	
Parents’ negative beliefs related to vaccination	217	65.4
Hours of vaccination are incompatible with parents’ schedule	121	36.4
Health workers do not ask of children’s vaccination status	19	5.7
Health workers do not review children’s vaccination cards or vaccination status	22	6.6
False contraindications for vaccination by health workers	36	10.8
Distance from the vaccination site	146	44.0
All of the above	39	11.7
**Completing vaccination registers delays vaccination**	**332**	
Agree	104	31.3
Disagree	228	68.8
**What instructions do you give caregivers when you give them a new vaccination card?** ^**1**^ ^,^ ^**2**^	**185**	
Keep the card safe	176	95.1
Bring this card to all visits to the health facility	91	49.2
Bring this card only when you come for vaccinations	104	56.2
No instruction given	1	0.5
Others	7	3.8
**There is sufficient staff offering immunization services at this health facility** ^**2**^	**185**	
Agree	69	37.3
Disagree	116	62.7
**There are enough vials of vaccine for all patients in need** ^**2**^	**185**	
Agree	174	94.1
Disagree	14	7.6
**There are enough materials for vaccination** ^**2**^	**185**	
Agree	181	97.8
Disagree	4	2.2

^1^ Respondents were allowed to select multiple responses.

^2^ Only asked of health workers who administer vaccines as part of their job.

### Knowledge, attitudes, and practices

Two-fifths (41.0%; 136/332) of the health workers believed their knowledge of vaccination was insufficient. Additionally, mor than half (57.5%; 191/332) of health workers said they were concerned about and feared AEFI. During the FGD, one health worker expressed her concerns as follows: “*Not all babies are being vaccinated*. *When I gave birth to my 1*^*st*^ *born after being injected as a treatment to an illness*, *my baby’s leg turned and since then*, *whenever my baby is sick*, *I will not allow him to be injected”* [FGD1_HW_01].

When asked about valid contraindications to vaccination, only a third of health workers (32.2%; 107/332) were able to correctly identify pneumonia and other serious diseases as true contraindications from a multiple-choice, multi-select list of options (which also included local reaction to a previous dose, low-grade fever, and seizures under medical treatment). This knowledge gap was corroborated in the FGD, with some health workers mentioning high fever, pre-term birth, jaundice as contraindications. “*Yes*, *at times*, *when as a health worker*, *you may be looking at a baby that is clinically looking ill*. *So*, *in such a situation*, *you don’t go ahead and immunize the child or maybe you touch a baby and you see that this baby is warm and you now check the temperature*, *it’s 38*^*°*^*C*, *definitely*, *you would know that this baby is running a fever*. *So*, *by the time you add immunization to it*, *the baby may not be able to tolerate whatever or the type of fever*. *It may even get to the extent of about 40*^*°*^*C and the baby convulses*, *which is not very good for the baby”* [FGD 3_HW_07].

More than half of health workers stated their regular duties included administering vaccines (55.7%; 185/332). Among these, the majority (62.7%; 116/185) disagreed that the health facility was adequately staffed for immunization but agreed (94.1%; 174/185) that there were enough vaccine vials for all. To corroborate the inadequacy of staff, one IDI participant noted “*We are short on staff*, *we don’t have enough of them*. *We only have two government employed staff here now*, *those staff you are seeing are PBF [sic]* [*Performance Based Financing] staff*, *like borrowed staff which we pay a sum of N10*, *000 monthly*. *Imagine such a ridiculous amount being paid to them*. *It is the major problem we are facing here*” [IDI_05].

### Perceived reasons for MOV among caregivers and health workers

From the caregivers and health workers’ FGDs and IDIs, as well as the brainstorming sessions, insights into the perceived reasons for MOV were identified. The perceived reasons identified for MOV were in three categories; reasons related to inadequate knowledge or poor attitude among health workers, those related to caregivers, and those related to the organization of the health system. One health worker FGD participants said, *“Maybe the child has lost weight or maybe when we see the card that they use to give us*, *you can see the weight of a child and if the child is less than 2kg*, *I think this can also hinder the child from being immunized*” [FGD5_HW_01]. **“***When a child delivered is underweight*, *such child is first taken care of before immunization would be given*” [IDI 13]. One caregiver said **“***What I think causes it is that they don’t have enough equipment and then they don’t have enough vaccines for children because during the time they go and bring the vaccines*, *sometimes they tell the mother of nine months [sic] [old] children*, *that if they are not many*, *they won’t be able to open the vaccine because there is a standard number that should be given at once*. *But*, *what I feel is that if there is enough*, *they won’t have to wait for children to be many before they can open it up*. *So*, *if they have these [sic] [vaccines] and enough equipment on standby*, *there won’t be a child that comes here that will not be vaccinated*” [FGD2_CG_06]. A caregiver mentioned overcrowding and the insufficient number of health workers as a reason for MOV, “*… you can get to the immunization center and the children that are there for immunization are up to 500*, *and we have just six health workers and this will actually jeopardize the integrity of the service of the health workers*, *this can actually lead to people missing their vaccination*” [FGD3_CG_05].

Health workers mentioned the organization of sessions as a reason for MOV *“If a mother brought her child to the center on a day that the center does not give immunization*, *such mother would be encouraged to bring her child on the next immunization day*. *Or*, *if there is another health center close by that is giving immunization*, *I will arrange for a motorcycle to take the mother to such clinic because if you ask the mother to go*, *she might not go*” [IDI_08].*“Why I think the child could possibly miss vaccination is this; Wednesday is the day for routine immunization in this health facility*, *but if the child should come here on Monday*, *there is every possibility for the child to miss being immunized*” [FGD5_HW_05].

Non availability of vaccine storage at facilities was also mentioned by health workers. “*We always go to the central store to collect vaccines*. *Had it been that we have our own refrigerator that we can keep vaccine*, *there won’t be a need going to that place*. *Because the idea of immunization now is when you see a child*, *open it*, *less emphasis has been placed on wastage*. *So*, *we are supposed to have this thing [sic] [own refrigerator]*, *it will ease the stress involved since we do not have a means of transportation*. *Moving around could be hazardous and anything can happen*. *So if we have our something [sic] [own vaccine] stock here inside our health center*, *it is preferable*” [IDI_13].

### Perceived solutions to reduce MOV among caregivers and health workers

Respondents called for a restructuring of the scheduling of vaccination at health facilities to make it more flexible so that children can receive vaccinations when due. “*I don’t know if it is possible to look at the schedule of vaccines*, *if the schedule is something that can be amended or restructured such that babies can always get their vaccines whenever they come around*, *not necessary at three months or a particular day of the week*” [CG_FGD_03].

Health workers recommended the recruitment of more staff, quality assurance systems, and in-service training for staff. “*The Ministry should employ more staff and pump more money for this vaccine [sic] [provide more funds for vaccination]*.” [FGD_HW-12] *“Encouraging the staff/health worker administering the vaccines will help us to work more*. *It is training*, *not necessarily money*. *Imparting more training*, *advance knowledge will further spur us to wanting to put it into practice*, *you want to see the reality*, *and you want to work more”* [FGD_HW_06].

Some health workers recommended equipping health facilities with storage devices. “*First thing is that each health center should have vaccine storage like a refrigerator*, *it is very compulsory*. *The SDD [sic] [Solar Direct Drive Vaccine Refrigerator]*. *Every government health center must have it because most of the time when people go to the farm*, *and they have something affecting them or wound*, *what they will think about is taking tetanus drug [sic] [vaccination] and they will rush to the health center and if the health center does not have it at that time*, *we may lose the client*. *So due to that*, *solar freezer or power or vaccines must be in health center 24 hours”* [IDI_HW_04].

## Discussion

The prevalence of MOV in Ondo State was found to be 33%. Recent MOV country assessments in the Americas and African regions of the WHO have shown that between 23% to 96% of eligible children who visited a health facility for vaccination or medical care, left the health facility without receiving the vaccine doses that they needed [6]. Similar to children in Ondo State, these children are already being reached by health services and not necessarily “hard-to-reach” or underserved populations. Missing the opportunity to vaccinate these children, when they are already present at the health facility on a vaccination day, is of concern and warrants interventions to reduce these MOV.

The occurrence of MOV in Ondo State was mainly among those who were at the health facility for a non-vaccination visit, with the highest prevalence of MOV among those who had medical consultations, followed by those who accompanied their caregiver and those who were brought for healthy child visits. These findings are consistent with other recent MOV assessments from Chad, Malawi, Kenya and Timor Leste [9,10,18]. If vaccinations or referrals are not provided during sick visits, there is a substantial risk that children will not return for immunizations. In our setting, the lack integration and of policies ensuring vaccination status checks are carried out outside of vaccination session’s sites may be responsible for the high MOV at non-vaccination visits we observed. Even during vaccination visits, vaccination status checks were inadequate and resulted in 7% MOV. This proportion is lower compared to other countries (Chad 34%, Timor Leste 32% and Malawi 23%) [9,10,18], but still shows that children are still not receiving all the vaccines they are eligible for. Studies have shown that ensuring that health workers screen vaccination documentation for vaccination status can greatly improve coverage rates and protect children [10]. Therefore, implementing vaccination status checks at all visits, can increase both timeliness and overall vaccination coverage. For this to be effective, however, all health workers will need to be engaged and trained. Experiences from other countries indicate that although health workers may request for the vaccination documentation, they may not actually review them for vaccination eligibility, but rather largely to record or verify demographic data [18]. Although a lower proportion of children () who came for vaccination experienced a MOV in our assessment.

With regards to dose-based MOV, our assessment found a higher prevalence of MOV among vaccines given to older children, particularly MenA and second dose of measles vaccines. This finding is consistent with other assessments which have also found an increase in MOV with age [5–18]. This can be an indication of a need to strengthen the second year of life platform (2YL), which can often be weak due to complacency and the reduced perception of the importance of vaccination beyond the first year of life [11,18]. In our context, measles second dose was introduced about 4 weeks before the assessment. Routine immunization intensification was used for about a week as a strategy to increase awareness, and to vaccinate eligible children with measles second dose and missed doses of other vaccines. If this strategy had been effective, we would have expected a lower proportion of MOV in the age group [10]. In addition, our findings demonstrated that opportunities to vaccinate with the measles vaccine were more frequently missed compared to the pentavalent or oral polio vaccine. This is consistent with other reports, and it likely due to the measles vaccine provided as a ten-dose vial, which must be used within six hours once reconstituted. Hence, this may amplify concerns among health workers about vaccine wastage [10]. It is therefore important that health workers’ training includes the EPI policy of opening a multi-dose vial, even for one eligible child. Additionally, having measles vaccine in smaller vials could also reduce MOV [21–23].

The reasons identified for MOV in our study can be grouped into three categories: reasons related to health workers, those related to caregivers, and those related to the organization of the health system. These reasons are consistent with MOV assessment findings reported in other settings [9,17,18]. We also found fear of AEFI, and difficulties accessing health services were associated with MOV and untimely vaccination.

In this assessment, timeliness of vaccination among children with documented vaccination history varied by vaccine and dose, with later vaccine doses recommended in a series being given in a less timely manner, which is consistent with other MOV assessments [9,18]. It has been documented that reducing MOV will also improve the timeliness of vaccination, improve the efficiency of health service delivery in general, and promote synergy between treatment services and preventive programs at the health facility level [7]. Besides, improving the timeliness of vaccine administration and reducing MOV, it can improve immunization coverage thereby reducing morbidity and mortality [12].

Diverse strategies have been proposed to promote timely vaccination and reduce MOV [24]. Some of these are similar to suggestions the health workers and caregivers proposed during the qualitative and quantitative interviews. The magnitude and causes of MOV may vary by country, and in different areas within a country. Therefore, conducting similar MOV studies may help authorities identify reasons for MOV and tailor solutions to their context. The need for service integration between the vaccination and non-vaccination services is demonstrated by the wide gap between the prevalence of MOV among caregivers who visited health facilities for vaccination and non-vaccination services. Standard clinical practices should include vaccination status checks at every health service encounter. Studies also indicate that caregivers seeking treatment for sick children in settings that do not normally provide vaccinations, such as pharmacies, marketplaces, and traditional healers [10] may provide opportunities to screen for vaccinations, remind caregivers of missed vaccinations, and provide instruction on where to get vaccination services. However, this may be challenging to implement, particularly if the caregiver does not bring vaccination documentation to every health service contact. Therefore, it is important that National immunization policies and guidelines are revised to clearly articulate the actions needed by health workers. Revisions may include immunization screening at every health service encounter, specific lists of co-administered vaccinations, opening vials for every child eligible for a missing vaccine and clarifications of valid and invalid contraindications [10]. Additionally, caregivers need to be encouraged to retain and bring vaccination documentation to every health service encounter.

The brainstorming session provided the opportunity to develop a work plan of activities to reduce MOV for the State. At the debrief with the leadership of the OSPHCB and immunization partners, the summary of the objectives of the assessment, the fieldwork process and the preliminary results and recommendations, as well as the proposed action plan, were provided. This was endorsed for implementation by the leadership of the State Primary Health Care Board. An evaluation of the impact of the implementation has been incorporated into the plan, and a post assessment is expected 8–12 months following implementation of the proposed action plan. However, due to COVID-19, these plans have been delayed.

### Limitations and opportunities

We recognize the limitations as well as the opportunities of the MOV methodology employed for this MOV assessment. The assessment led to a participatory process of investigation about the importance of MOV and the development of strategies to reduce their occurrence. Although the study is not a rigorous statistical measurement of the prevalence of MOV, the WHO strategy provided field-friendly and practicable tools which can lead to effective public health action. In addition, this was a health facility based study, conducted in a purposive sample of facilities, those offering routine immunization, in only three out of 18 LGAs of Ondo State, on a single day per facility or multiple of facilities. It gives an indication of how common MOV are on the assessment day, as well as qualitative insights into the causes of MOV. However, the assessment was not designed to give a representative estimate of MOV frequency, and we did not account for clustering in both the design and analysis of the assessment, even though children are nested within health facilities. However, it should be noted that accounting for the clustering would impact standard errors only, and our analysis presented descriptive percentages for the MOV estimate. In addition, no meaningful comparison could be made between the government and private health facilities, because a very few number of private facilities are providing routine immunization services in Ondo State. The analysis of MOV was based on a subsample of children with valid documentation of dates of births and vaccination dates as evidenced by the vaccination documentation or health facility register. However, the characteristics, attitudes, and knowledge of caregivers with valid documentation of the dates of births and vaccinations and those without, were not statistically different, indicating minimal or no selection bias in the subsample used for MOV estimation in this study.

## Conclusion

The MOV assessment in Ondo State has shown that one in three children eligible for one or more vaccines and who visited the health facilities on the day of the assessment experienced a MOV. The proportion of MOV was higher among children who were at the facilities for non-vaccination visits, despite having had contact with the health system. These MOV may have occurred due to a lack of institutionalized screening of vaccination documentation by health workers, especially those providing non-vaccination services, caregivers not bringing, vaccination documentation for non-vaccination services, non-availability of vaccination services on all days of the week, lack of an appropriate referral system between non-vaccination and vaccination services within the same facility, shortage and maldistribution of health workers and gaps in knowledge among available health workers on proper screening of vaccination documentation, multidose vial policy, their concern on vaccine wastage rates, AEFI, and attitudes to clients. Some recommendations made, include the adoption of screening vaccination documentation at every health contact, conduct of daily vaccinations at health facilities, training of health workers on identified knowledge/attitude gaps and caregivers sensitization. These recommendations were developed into an actionable work plan, which will be evaluated at regular intervals for its impact.

## Supporting information

S1 File Compressed/Zip file data repository including qualitative and qualitative data tools and validated data. (ZIP)
