# Correction: Missed opportunities for vaccination among children aged 0–23 months visiting health facilities in a southwest State of Nigeria, December 2019

**DOI:** 10.1371/journal.pone.0325918

**Published:** 2025-06-02

**Authors:** Akinola Ayoola Fatiregun, Laura Nic Lochlainn, Lassané Kaboré, Modupeola Dosumu, Elvis Isere, Itse Olaoye, Francis Adegoke Akanbiemu, Yetunde Olagbuji, Rosemary Onyibe, Kofi Boateng, Richard Banda, Fiona Braka

The second author’s initials appear incorrectly in the citation. The correct citation is: Fatiregun AA, Nic Lochlainn L, Kaboré L, Dosumu M, Isere E, Olaoye I, et al. (2021) Missed opportunities for vaccination among children aged 0–23 months visiting health facilities in a southwest State of Nigeria, December 2019. PLoS ONE 16(8): e0252798. https://doi.org/10.1371/journal.pone.0252798
